# IL-8/CXCR1/2 signalling promotes tumor cell proliferation, invasion and vascular mimicry in glioblastoma

**DOI:** 10.1186/s12929-018-0464-y

**Published:** 2018-08-08

**Authors:** Ira Sharma, Avninder Singh, Fouzia Siraj, Sunita Saxena

**Affiliations:** 1 National Institute of Pathology, Safdarjung Hospital Campus, Room No. 610, 6th floor, Ansari Nagar, New Delhi, 110029 India; 2 Symbiosis International University, Pune, India

**Keywords:** IL-8, IL-8-CXCR1/2, Glioblastoma, Vascular mimicry, GBM progression

## Abstract

**Background:**

Glioblastoma multiforme (GBM) is one of the lethal malignant tumors of the central nervous system. Despite advances made in understanding this complex disease, little has been achieved in improving clinical efficacy towards it. Factors such as chemokines play important role in shaping the tumor microenvironment which in turn plays a significant role in deciding course of tumor progression. In this study, we investigated the role of chemokine IL-8 in glioblastoma progression with particular emphasis on immunomodulation, cellular proliferation, invasion and vascular mimicry.

**Methods:**

Role of IL-8 in GBM immunology was determined by correlating the expression of IL-8 by immunohistochemistry with other immune cell markers such as CD3 and CD68. Effect of high IL-8 expression on overall survival, the difference in expression level between different GBM subgroups and anatomic structures were analyzed using other databases. Two GBM cell lines –U-87MG and LN-18 were used to study the impact of targeting IL-8-CXCR1/2 signalling using neutralizing antibodies and pharmacological antagonist. Reverse transcriptase–polymerase chain reaction and immunocytochemistry were used to determine the expression of these axes. Impact on cell viability and proliferation was assessed by MTT, proliferation marker-ki-67 and clonogenic survival assays. Multicellular tumor spheroids generated from GBM cell lines were used to study invasion in matrigel.

**Results:**

Weak Positive correlation was observed between IL-8 and CD3 as well as between IL-8 and CD68. High IL-8 expression in GBM patients was found to be associated with dismal survival. No significant difference in IL-8 expression between different molecular subgroups of GBM was observed. In vitro targeting of IL-8-CXCR1/2 signalling displayed a significant reduction in cell viability and proliferation, and spheroid invasion. Furthermore, the presence of CD34-/CXCR1+ vessels in GBM tissues showed the involvement of IL-8/CXCR1 in vascular mimicry structure formation.

**Conclusion:**

These results suggest a direct involvement of IL-8-CXCR1/2 axes in GBM progression by promoting both cell proliferation and invasion and indirectly by promoting neovascularization in the form of vascular mimicry.

**Electronic supplementary material:**

The online version of this article (10.1186/s12929-018-0464-y) contains supplementary material, which is available to authorized users.

## Background

Glioblastoma multiforme (GBM) is the most recalcitrant primary brain tumor. The aggressive behavior of this tumor is due to rapid local invasion and resistance to therapy. It is known to be the most hypoxic and highly vascularized tumor exhibiting enormous molecular heterogeneity which poses serious challenges in designing effective treatment strategies for all GBM patients resulting in poor success with current treatment modalities [1].

Tumor microenvironment harbors various cell types and plays a dominant role in deciding course of tumor progression. Within this microenvironment tumor cells inhabiting different niches behave differently. For instance, tumor cells thriving in hypoxic area release factors which promote neovascularization [2], similarly tumor cells present in well-vascularized areas in brain-enriched with intact vessels and ample oxygen release factors which promote aggressive proliferation and invasion [3]. A factor such as vascular endothelial growth factor (VEGF) is extensively studied in malignant gliomas and thought to be playing a pivotal role in angiogenesis and vascularization in these tumors. Hence, VEGF became a major target of anti-angiogenic therapy [4]. However, limited success with bevacizumab (humanized anti-VEGF antibodies) as first-line treatment in recurrent GBM patients has made it imperative to look for other factors of angiogenesis. Recent studies have shown the role of chemokines in different aspects of tumor growth in various cancers including GBM [5]. These molecules may be secreted by tumor cells or other cell types present in tumor microenvironment regulating tumor immune response, vasculogenesis and cellular proliferation [6]. One such chemokine is CXCL8 which is a known angiogenic factor and has also been shown to exert a mitogenic effect in cancers such as colon cancer, prostate and ovarian cancer [7, 8].

CXCL8 also known as IL-8 (Interleukin-8) is CXC chemokine with the pro-inflammatory property. IL-8 gene transcription results in 99 amino acids long protein which is further processed to yield either 77 amino acid protein which is found in non-immune cells or 72 amino acids long protein produced by immune cells such as monocytes and macrophages [8, 9]. It is not normally expressed by non-cancerous cells however the expression of IL-8 is inducible by various stimuli, varying from inflammatory signals, stress signals from environment or chemicals to hormone-induced signals (such as estrogens and androgens) [9, 10]. In contrast, many tumor cells have been shown to constitutively express this chemokine. IL-8 exerts its biological effect through CXCR1 and CXCR2 receptors which are G-protein coupled receptors [11, 12]. This chemokine acts as a multifunctional cytokine which can mediate tumor cell proliferation, migration, and invasion in autocrine or paracrine mode. Dwyer et al. recently showed IL-8 involvement in enhancing vascular permeability in GBM [5]. Further expression of this chemokine has also been shown in cancer stem cells in various cancers including GBM [13]. Previously, we had studied differential gene expression pattern of chemokines between low-grade astrocytoma (grade II) and High-grade astrocytoma (Grade IV, GBM) which led to the identification of key pro-gliomagenic chemokines. IL-8 was among the top chemokines which were up-regulated in GBM as compared to low-grade astrocytoma showing more than 4 fold gene up-regulation. A comparison with other datasets ranked IL-8 as most frequently up-regulated chemokine among those selected chemokine genes in the GBM datasets available on Oncomine [14].

In the present study, we aimed to elucidate the role of IL-8 in GBM biology wherein we investigated its role in immune cell infiltration, GBM cell proliferation, invasion, and vascularization.

## Methods

### Tissue samples

Patient sample collection was done after taking approval from the institutional ethical committee of Safdarjung Hospital. Each tissue sample was fixed in 10% buffered formalin which was later processed and embedded in paraffin. Formalin-fixed paraffin embedded (FFPE) sections were evaluated independently by two pathologists and a consensus in the diagnosis, as well as its WHO grade, was achieved. A total of 48 WHO grade IV astrocytoma (glioblastoma) and five non-neoplastic brain tissue as control were included in this study.

### Immunohistochemistry

Antibodies against IL-8 and CXCR2 were purchased from Biorbyt (UK). Anti-CXCR1 was purchased from Boster Bio (USA). Anti-CD3, EnVision polymer detection system, anti-CD68, and anti-CD34 were purchased from Dako, Agilent Pathology solutions, Denmark. A standard immunohistochemical (IHC) technique was followed as described previously [15]. A semiquantitative analysis was done using IHC scoring of 0, 1 and 2. A ‘0’ score indicated no scoring, ‘1’ indicated < 30% cells showing immunostaining and ‘2’ indicated > 30% cells showing immunostaining. For correlation study between IL-8, CD68 and CD3 positivity, total percentage positivity was calculated in five different microscopic fields and average percentage was calculated for each case.

### RNA extraction and RT-PCR

Total RNA was extracted from two GBM cell lines using Qiagen RNeasy mini kit (Qiagen, Germany) as per the manufacturer’s guidelines. cDNA conversion of 2 μg RNA was done using High-Capacity cDNA Reverse Transcription Kit (Applied Biosystems, Thermo Fisher Scientific, US). A manufacturer-defined protocol was followed for cDNA conversion. RT-PCR was performed using Fermentas Taq DNA polymerase (standard PCR components other than primers were provided in this kit). Briefly, 200 ng of cDNA template was amplified in 36 cycles, the PCR cycle consisted of 30 s at 94 °C, 30 s at (56 °C–63 °C, standardized for each gene) and 1 min at 68 °C. The expected product length and primer sequence are given in Additional file 1: Table S1.

### Immunocytochemistry

Immunocytochemistry was performed on GBM cell lines grown on poly-l-lysine coated coverslips. Cells were fixed with 4% paraformaldehyde for 15 min and washed in PBS (Phosphate Buffer Saline) for 5 min. Cells were then permeabilized in PBS with 0.1% Tween 20 for 10 min. Following this protein block was added to block non-specific binding sides for 15 min. Excess protein block was removed and primary antibody was added and incubated at room temperature under the humidified condition for 1 h. Unbound antibodies were removed by washing in PBS for 5 min. For visualization secondary FITC conjugated antibody (Dako, Agilent Pathology solutions, Denmark) was added so as to cover the coverslip and incubated at room temperature for another 1 h under same conditions. Further washing was done and counterstaining was done with DAPI (Fluoroshield, Sigma Aldrich, US) and mounted over glass slides. Fluorescent microscopy was done and images were captured.

### Cell lines and reagents

Two GBM cell lines U-87 MG and LN-18 were procured from National Centre For Cell Science (NCCS), Pune. U-87 MG cell line was maintained in Eagle’s Minimum Essential Medium (MEM) (Sciencell, Research Laboratories, US) supplemented with 1% NEAA(Non-essential amino acids, from Gibco, Thermo Fisher Scientific, US), 1 mM Sodium pyruvate (Gibco, Thermo Fisher Scientific, US), 1% Penicillin / Streptomycin /Amphotericin B (BioBasic, US) and 10% Fetal Bovine serum (FBS) (Gibco, Thermo Fisher Scientific, US). LN-18 was maintained in Dulbecco’s Modified Eagle Medium (DMEM) with 4.5 g Glucose per liter (Gibco, Thermo Fisher Scientific, US), 1 mM Sodium pyruvate, L-Glutamine 1.5 g per liter of Sodium bicarbonate), 1% Penicillin / Streptomycin /Amphotericin B and 10% FBS. Serum-free media was used for functional assays.

### Antagonist treatment and cell viability assay

For cell viability and proliferation, MTT assay was performed as per manufacturer’s guidelines (HPLC chemicals, India). Briefly, 3000 cells/100 ul media with 2% FBS were seeded in 96 well plate (Falcon, BD Biosciences). For antagonist treatment, serum-free media was added and cells were treated with reparixin-l-lysine (Apex Bio, US) for different durations (24, 48 and 72 h). 20 μl of MTT (3-(4, 5-Dimethylthiazol-2-yl)-2, 5-Diphenyltetrazolium Bromide) reagent was added into each well and formazan crystals were dissolved in 10% SDS in 0.01 N HCl. After 4 h absorbance was recorded at 570 nm using a 96-well plate reader.

### Ki-67 labelling

Approximately 50,000 cells were grown on poly-l-lysine coated coverslips in 24 well plates. After overnight incubation, cells were treated with 10 μM reparixin-l-lysine. Immunocytochemistry with ki-67 antibody was performed after 24 h of treatment. Proliferation index was calculated using ImageJ 1.X software. Total number of cells was calculated based on DAPI staining in five different microscopic fields and percentage of ki-67 positive cells was calculated in each of these fields using following formula –

$$ \mathrm{Percentage}\kern0.5em \mathrm{of}\kern0.5em \mathrm{proliferating}\kern0.5em \mathrm{cells}\kern0.5em =\kern0.5em \frac{\mathrm{Total}\ \mathrm{number}\ \mathrm{of}\ \mathrm{ki}\hbox{-} 67\ \mathrm{positive}\ \mathrm{cells}\ \mathrm{in}\kern0.5em 5\kern0.5em \mathrm{different}\kern0.5em \mathrm{microscopic}\kern0.5em \mathrm{fields}\kern0.5em \times \kern0.5em 100}{\mathrm{Total}\kern0.5em \mathrm{number}\kern0.5em \mathrm{of}\kern0.5em \mathrm{cells}\kern0.5em \mathrm{in}\kern0.5em \mathrm{different}\kern0.5em \mathrm{microscopic}\kern0.5em \mathrm{fields}} $$

### Neutralization assay

Approximately 3 × 10^3^ cells were seeded in 96 wells plate. Cells were allowed to attach to the surface overnight. Anti-CXCL8 (R&D systems) (1 μg/ml), Anti-CXCR1 (R&D systems) (1 μg/ml) and Anti-CXCR2 (R&D systems) (1 μg/ml) were added to each well except control either alone or in combination. Control was treated with Goat IgG (R&D systems). After 24 h cell viability was tested by MTT as described above.

### Clonogenic assay

Approximately 500 cells were seeded in 6 wells plate. After overnight incubation cells were treated with Reparixin-l-lysine at 10 μM concentration. Media was replaced every 3 days along with drug treatment. After 15 days colonies were stained with Coomassie brilliant blue. Colonies were counted using Image J 1.X software.

### Spheroid invasion assay

This assay was performed as described previously [16]. Briefly, 1 × 10^3^ cells were seeded in ultra-low attachment (ULA) 96-well round bottom plates (Nunclon sphere, Thermo scientific, US) in complete media. After 4 days drug (Reparixin-l-lysine, 10 μM) was added, followed by 100 μl matrigel matrix (BD Biosciences, US) in each well. After 1 h, 100 μl complete media was added to each well. Images were acquired after 24 h, 48 h and 72 h. Image analysis and invasion were performed using Image J 1.X software.

### Statistical analysis

Statistical analysis was done using SPSS v17.0, Chicago, IL, USA. *P* values were calculated using Student’s t-test with two-tailed distribution and value of less than 0.05 was considered statistically significant. Pearson’s correlation coefficient and one way ANOVA was applied wherever required.

## Results

### Effect of IL-8 expression on immune cell infiltration

**Fig. 1:**
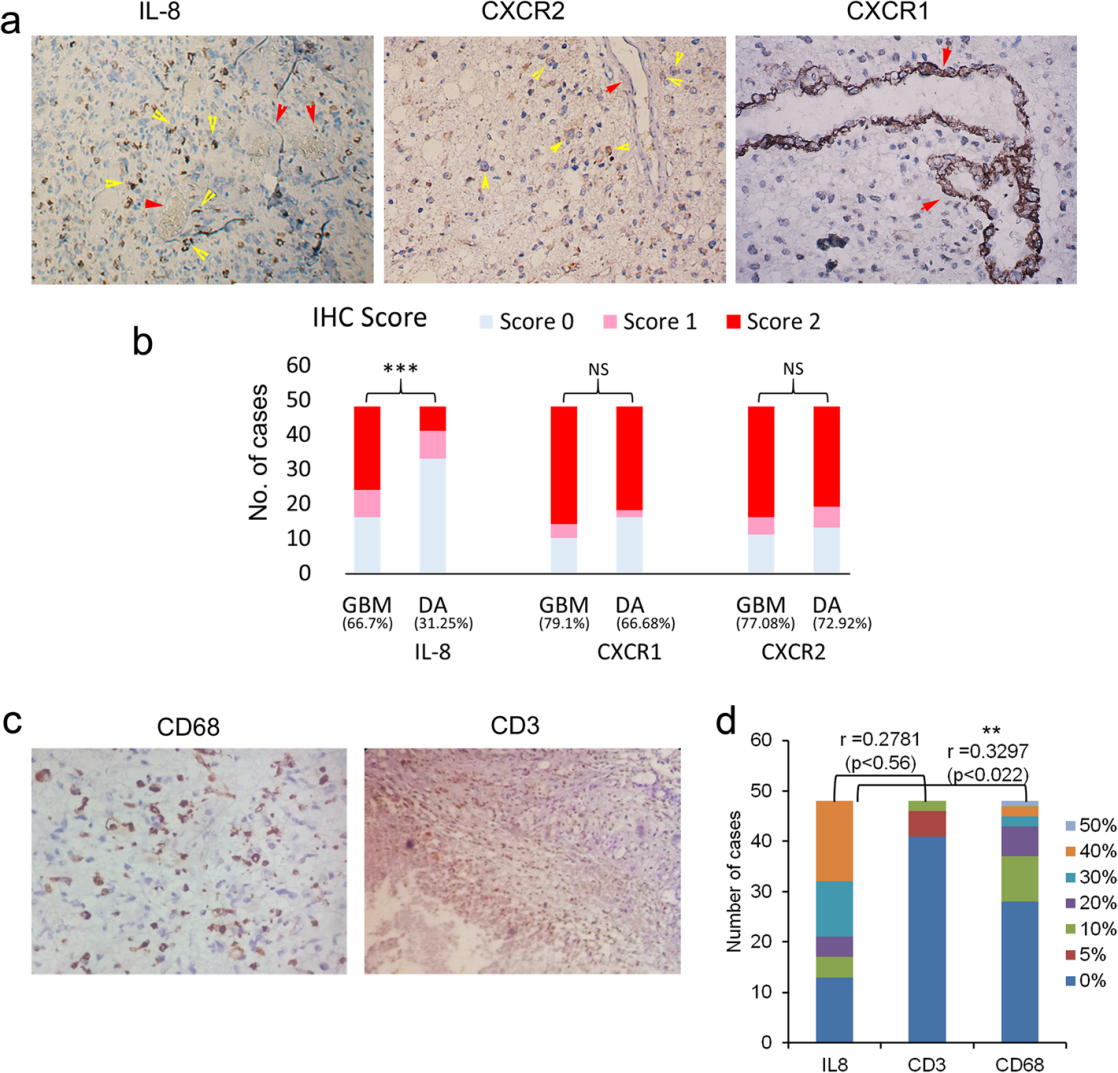
Effect of IL-8 expression on immune cell infiltration. **a** Immunohistochemical expression of IL-8, CXCR1, and CXCR2 in FFPE tissues of GBM. Image resolution was × 200. Yellow arrows indicate staining in tumor cells. Red arrows indicate tumor vasculature which is showing positive staining only in CXCR1. **b** Stacked bar graph showing IHC score in GBM and Diffuse Astrocytoma (DA) in 48 cases each. Fisher’s exact test was used to analyse the statistical difference in immunohistochemical staining of IL-8, CXCR1 and CXCR2 between the two groups. *** indicates *p* < 0.001, NS=Non-significant. Total percentage positivity for individual protein in each group is given below each bar. **c** Immunohistochemical expression of CD68 and CD3 in FFPE tissues of GBM. The image resolution was at × 200 for CD68 and × 100 for CD3. **d** Stacked bar graph showing percentage positivity of IL-8, CD3, and CD68 in 48 GBM cases. Percentage positivity ranged between 0 and 50% in each group. Different colors in each bar graph represent the number of cases showing respective percentage positivity. r values indicate Pearson’s correlation coefficient calculated between IL-8 and CD3 and between IL-8 and CD68. The statistically significant positive correlation was observed between IL-8 and CD68. ** indicates *p* < 0.01

Previously we had done gene expression study of an entire panel of chemokines and their receptors between low-grade astrocytoma (Diffuse Astrocytoma) and high-grade astrocytoma (GBM), followed by gene ontology analysis and comparison with other databases which highlighted IL-8 as the most frequently upregulated chemokine in GBM. Further, protein localization study performed on GBM and diffuse astrocytoma FFPE sections using immunohistochemistry established expression of IL-8 in GBM cells and expression of its receptor CXCR1 primarily in tumor-associated vessels and in few cases in tumor cells as well [14]. Furthermore, expression of its alternate receptor CXCR2 was observed in tumor cells only (Fig. 1a). Statistically significant difference in immunohistochemical staining was observed only for IL-8 between GBM and diffuse astrocytoma (Fig. 1b). Since other ligands of CXCR2 and CXCR1 were not significantly upregulated in gene expression study, those were not pursued for protein localization.

Considering the results of gene expression and protein localization study, it is appropriate to say that IL-8 is the main ligand of CXCR1 and CXCR2 in GBM and that their activation and downstream signalling are dependent on IL-8 expression and binding.

IL-8 is known to be a pro-inflammatory chemokine which promotes trafficking of leukocytes at the site of inflammation and in the tumor microenvironment [17]. Hence to investigate its potential role as a recruiter or suppressor of immune cells, we studied the correlation between IL-8 expression and CD3 expression in 48 GBM cases and also compared the IL-8 expression and CD68 in same tissue samples (Fig. 1c and d). Pearson’s Correlation Coefficient (r) between IL-8 and CD3 was 0.2781 (*p* < 0.56) which is weak and statistically insignificant. On the other hand correlation coefficient between IL-8 and CD68 was 0.3297 (*p* < 0.022) which is again weak positive correlation but statistically significant.

Hence from these results it may be interpreted that increased IL-8 expression has no overall impact on lymphocytic infiltration (CD3 positive cells) which was very low ranging between 0 and 10% in GBM, while increased IL-8 expression appeared to be favoring microglial infiltration in GBM. This may have an immunosuppressive effect, depending on polar tilt (M1 or M2 polarization) of these infiltrating macrophages. However, weak positive correlation between IL-8 and CD68 expression does not make IL-8, a prime contributor in CD68 positive cell recruitment. This also implicates that immunomodulation is not the primary role of IL-8 in GBM biology.

### Comparison with other databases

**Fig. 2:**
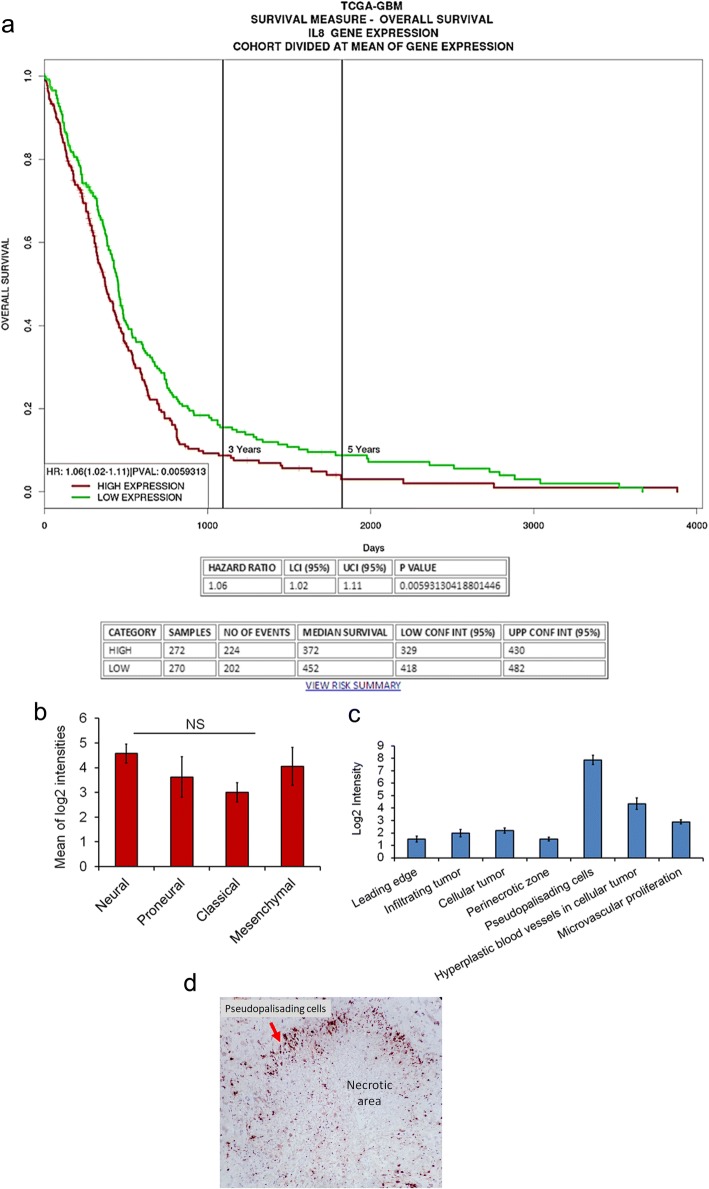
Comparison with other databases. **a** Kaplan-Meier survival graph plot to show the difference in survival between high IL-8 GBM group and low IL-8 GBM group of patients. Plot generated online using TCGA (The Cancer Genome Atlas) GBM datasets available on PROGgeneV2 (A prognostic database) (**b**) Bar graph showing mean log2 intensity of IL-8 probe in different GBM subgroups namely- neural, proneural, classical and mesenchymal. Error bar indicates standard deviation. One way ANOVA was used to analyze the statistical difference between the groups. NS indicates the non-significant difference. **c** Bar graph showing mean log2 intensity of IL-8 in different anatomic structures of GBM tissues. Error bar indicates standard deviation. **d** Immunohistochemical staining of IL-8 in GBM. The image was taken at × 200 resolution

To understand the impact of increased expression of IL-8 on overall survival of GBM patients, survival analysis of TCGA data of GBM involving 577 cases using PROGgeneV2 [18] was performed. Out of 577 cases, 270 cases exhibited increased IL-8 expression while 272 showed low expression of IL-8. The median survival of high IL-8 group was 372 days while the low IL-8 group had a median survival of 452 days. This indicates a significant association of increased expression of IL-8 in GBM patients with poor overall survival (Hazard ratio [HR] =1.06, 95% confidence interval [CI]: 1.02–1.11 and *p* < 0.0059) (Fig. 2a).

Further, we confirmed whether IL-8 expression pattern varies between four subgroups of GBM i.e. neural, proneural, classical and mesenchymal. To this end, we utilized the RNA seq data from IVY glioblastoma atlas project (http://glioblastoma.alleninstitute.org/rnaseq/search/index.html) [19, 20] which comprised of 37 cases. A comparison of *IL-8* gene expression between these four GBM subgroups displayed no significant difference *p* < 0.6397 (Fig. 2b). The difference was analyzed using one way ANOVA. Furthermore, we utilized the same database to identify differential gene expression pattern of IL-8 between different anatomic structures in GBM histological specimens. The anatomic structures analyzed were leading edge, infiltrating tumor cells, cellular tumor, microvascular proliferation, perinecrotic area and pseudopalisading cells around necrosis. Figure 2c represents the difference in mean log2 intensities between these structures. Maximum IL-8 expression was seen in pseudopalisading cells. This is similar to our observation of IL-8 immunoexpression where we also witnessed expression in pseudopalisading cells (Fig. 2d). However, in this data expression of IL-8 is also shown in hyperplastic blood vessels, while we did not observe expression of IL-8 in blood vessels. Figure 1b shows blood vessels negative for IL-8 which is highlighted with yellow arrows. Instead, we found expression of its receptor CXCR1 in these vessels (Fig. 1c).

Taken together, protein localization, survival analysis and gene expression analysis from other databases suggest the substantial involvement of this chemokine in GBM biology which needs further investigation involving functional characterization.

### In vitro targeting of IL-8/CXCR1/2 axes significantly impact tumor cell proliferation and survival

**Fig. 3:**
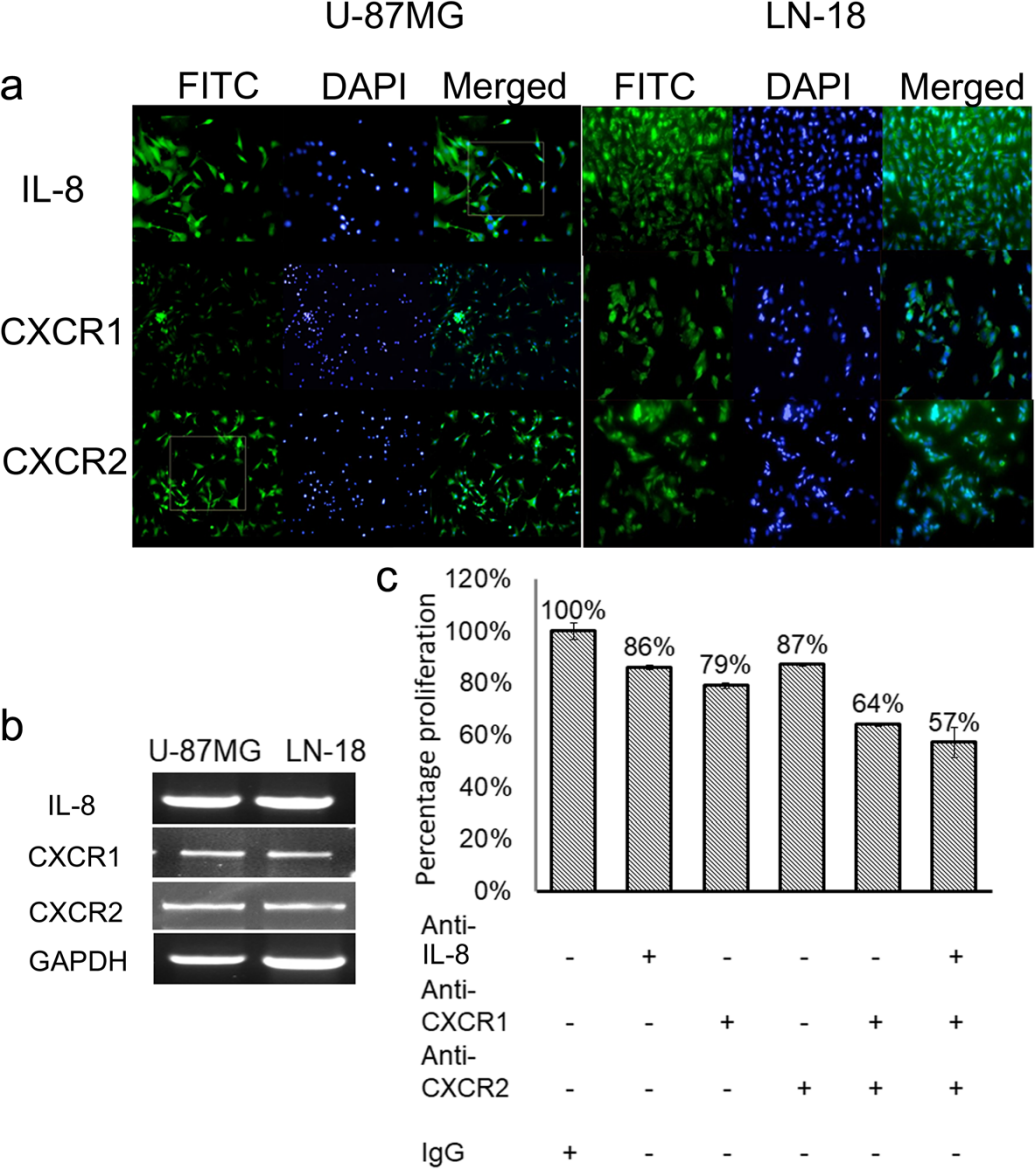
Expression of IL-8/CXCR1/2 in two GBM cell lines. **a** Immunocytochemistry (ICC) showing expression of IL-8, CXCR1, and CXCR2 in U-87MG and LN-18 cell lines. **b** RT-PCR of IL-8, CXCR1, CXCR2, and GAPDH in U-87MG and LN-18 cell lines. **c** Neutralization assay in U-87MG cell line. Effect on cell viability and proliferation was assessed by MTT after 24 h of neutralization with neutralizing antibodies against IL-8, CXCR1, and CXCR2. Error bar indicates standard error calculated from three independent experiments in triplicate

To investigate the exact biological role of IL-8/CXCR1/2 signalling GBM biology, first we checked the expression of IL-8, CXCR1 and CXCR2 at mRNA and protein level in U-87MG and LN-18 cell lines (Fig. 3a and b). IL-8 expression was high in both the cell lines while expression of its receptors was lower in comparison to IL-8. After confirming the expression we next sought to check the impact of neutralizing each protein individually and in combination, on tumor cell proliferation in U-87 cell line. For this, antibodies against IL-8, CXCR1, CXCR2 and isotype control IgG were added in each well containing U-87MG cells in different combinations (Fig. 3c). These antibodies neutralized their respective proteins thus impairing receptor-ligand binding. Due to this, an impairment in IL-8/CXCR1/2 signalling was expected. Since we suspected its involvement in cell proliferation and viability we performed MTT assay. Here we observed that individual blocking of IL-8, CXCR1, and CXCR2 by neutralizing antibodies led to a marginal decline in cell proliferation as assessed by MTT assay. However, blocking both the receptors led to an additive impact on proliferation and was maximum when all the three were neutralized simultaneously, suggesting an effective inhibition of these axes may directly impact tumor cell proliferation (Fig. 3c).

**Fig. 4:**
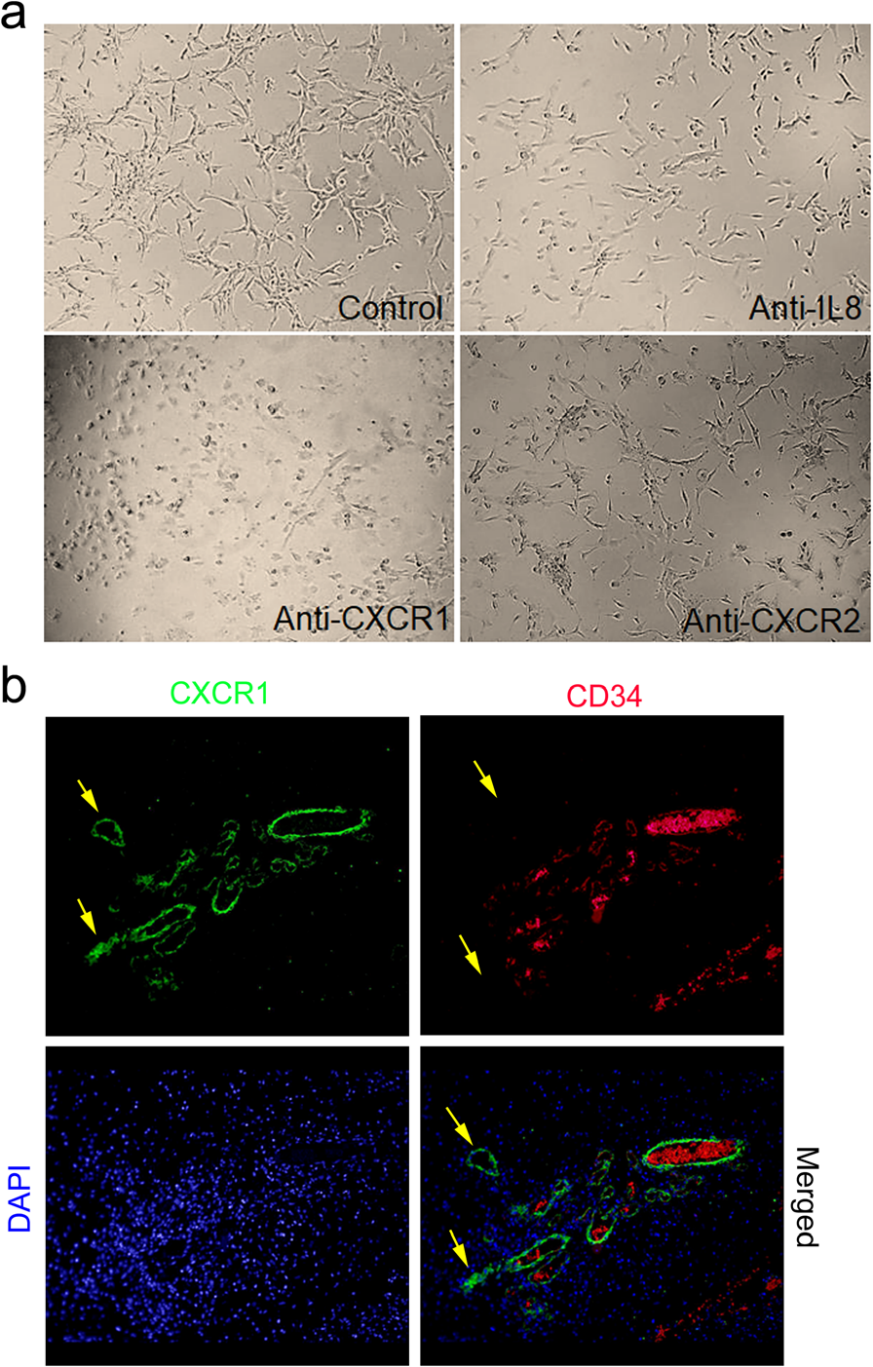
Vascular mimicry in GBM (**a)** Impact on the tubular structure in U-87MG cell line on neutralization with Anti-CXCL8, anti-CXCR1, anti-CXCR2. Image magnification × 100 (**b**) Double immunofluorescence staining of GBM tissue with Anti-CXCR1 (FITC, green) and anti-CD34 (Texas red). The yellow arrow indicates CXCR1^+^ /CD34^−^ microvessel. In CD34/Texas red staining yellow arrow indicates absence of two vessels which are present in CXCR1/FITC image. The images were taken at × 100

Also, we observed disruption of the tubular network in U-87MG cell line which is a peculiar characteristic of U-87MG cells exhibiting vasculogenic mimicry (VM) [21]. (Fig. 4a) Disruption was also observed when CXCR1 has neutralized alone, this in part supports our hypothesis that IL-8 via CXCR1 may promote angiogenesis in GBM. To get more clear evidence of the presence of CXCR1 on VM structures we performed double immunofluorescence staining of GBM tissue with anti-CXCR1 and anti-CD34 antibodies. Figure 4b shows the existence of both CD34+/CXCR1+ vessels as well as CD34-/CXCR1+ vessels highlighted by yellow arrows. Taken together, results of neutralization assay and comparison of double immunostaining of CXCR1 and CD34 in GBM tissues strongly suggest that IL-8-CXCR1 axis is not only involved in neovascularization but is also driving VM structure formation which has been considered an independent adverse prognostic factor in GBM [22].

**Fig. 5:**
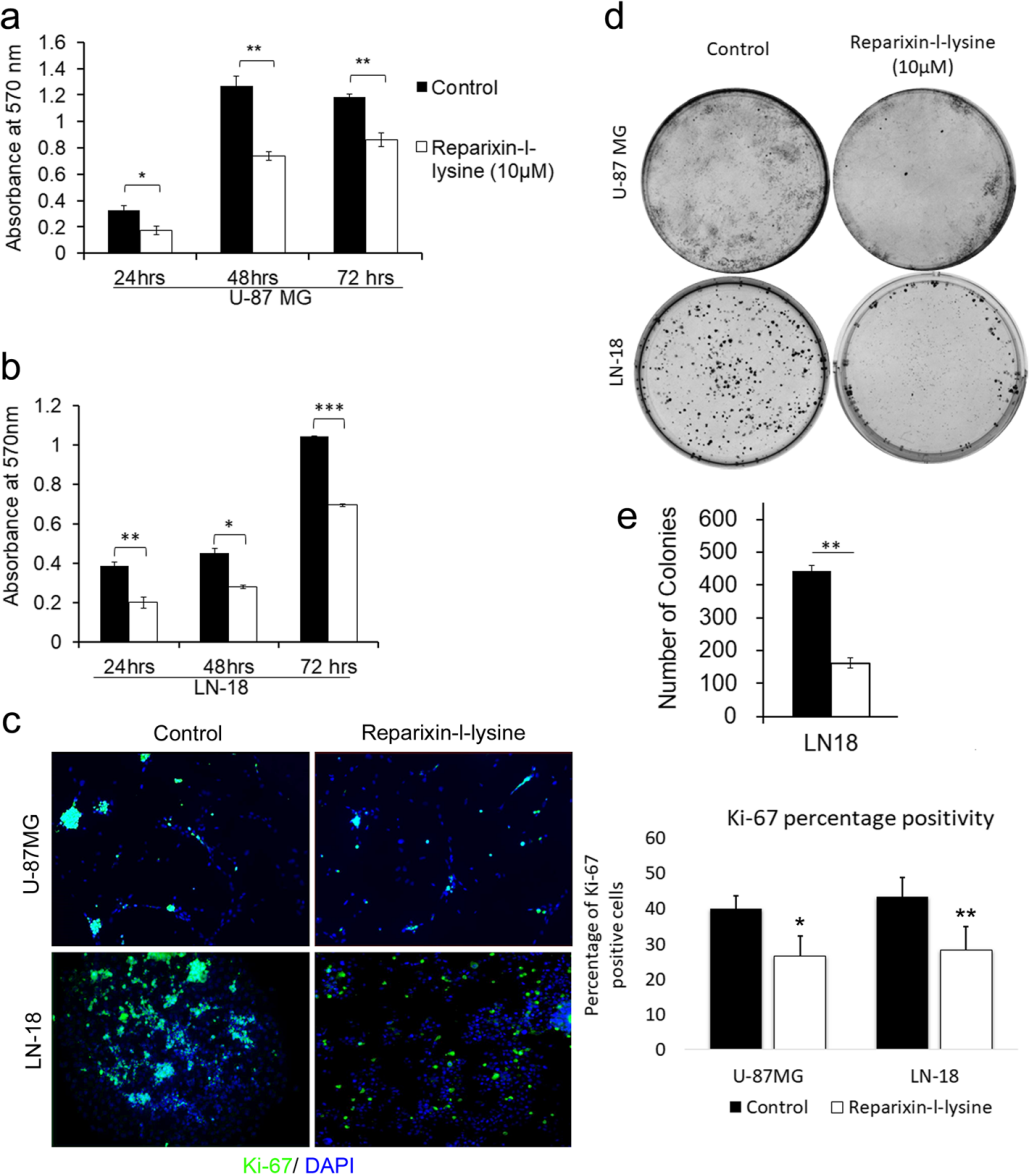
Cell proliferation and viability assays: (**a**) and (**b**) Bar graphs showing mean absorbance values obtained from MTT assay in U-87MG and LN-18 Cell lines respectively after treatment with 10 μM reparixin-l-lysine at different time intervals-24, 48 and 72 h. **c** Ki-67 labelling done by immunocytochemistry in U-87MG and LN-18 cells. The bar graph is showing percentage positivity of Ki-67 staining in both the cell lines. Student’s t-test was performed to analyze the statistical difference in percentage positivity of Ki-67 labelling between control and reparixin-l-lysine (10 μM) treated cells in both the cell lines. A *p*-*value of* less than 0.05 was considered statistically significant. **d** and **e** Clonogenic assay in U-87MG and LN-18 cell lines after treatment with Reparixin-l-lysine. Error bar indicates standard error calculated from three independent experiments in triplicate

After confirming the direct role of this axes in tumor cell proliferation we further examined the effect of chemical antagonist for CXCR1/2 – Reparixin-l-lysine. The IC-50 value was established at 10 μM and its effect at different time duration was assessed by MTT assay. Reparixin-l-lysine effectively abrogated tumor cell proliferation in 24 h and this effect persisted till 72 h, thereafter inhibitory effect diminished (Fig. 5a and b). Since MTT assay demonstrates both cell survival and proliferation, we analyzed the impact on proliferation alone by comparing ki-67 labelling of U-87MG and LN-18 cells under treated (10 μM Reparixin) and untreated (DMSO) conditions (Fig. 5c). Ki-67 labelling was measured using ImageJ. Ki-67 percentage positivity in control and reparixin-l-lysine treated groups of U-87MG and LN-18 cells were statistically analyzed using Student’s t-test. In the U-87MG control group, the percentage of proliferating cells was 40.14% while only 26.6% reparixin-l-lysine treated cells exhibited proliferation. The difference in proliferation percent between these two groups is statistically significant with *p* < 0.0184. Similarly, in LN-18 cell line, the average proliferating cells in control group was 43.45% while reparixin-l-lysine treatment significantly affected cell proliferation, wherein an average of 28.35% proliferation percentage was observed with *p* < 0.003. Further impact on cell proliferation was assessed by clonogenic assay where again a significant reduction in clonogenic survival was observed both in U-87MG and LN-18 cells treated with 10 μM Reparixin-l-lysine in comparison to control treated with DMSO after 15 days (Fig. 5d and e). However, quantitation was possible only in LN-18 cells as compact colonies were formed while U-87MG cells did not form compact colonies which made their quantitation infeasible.

In view of both neutralization of IL-8/CXCR1/2 axes with neutralizing antibodies and pharmacological blocking of CXCR1/2 with Reparixin-l-lysine which demonstrated a significant reduction in tumor cell proliferation, a direct and significant role of IL-8-CXCR1/2 axes in GBM growth cannot be discounted.

### CXCR1/2 antagonist inhibits the spheroid invasion in vitro in LN-18 and U-87 MG cell lines

**Fig. 6:**
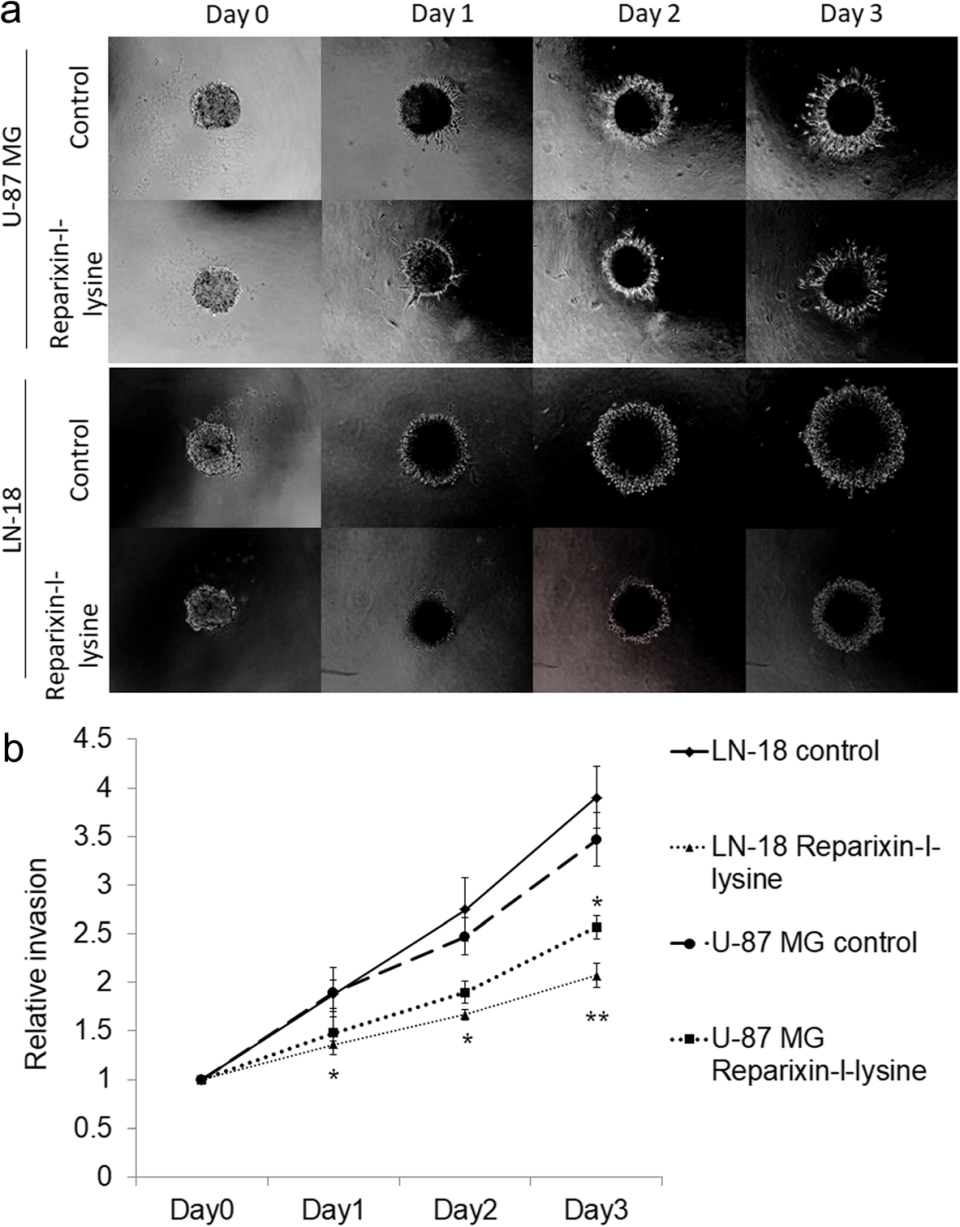
Impact on GBM cell invasion on targeting IL-8/CXCR1/2 (**a**) Spheroid invasion assay in U-87MG and LN-18 cell line (**b**) Graph showing relative invasion of U-87 MG and LN-18 cell lines. The statistically significant decrease in spheroid invasion was observed on day 3 in U-87MG cells treated with Reparixin-l-lysine (*p* < 0.05). In LN-18 statistically significant difference in spheroid invasion was evident from day 1with *p* < 0.05, which became even more prominent on day 3 with *p* < 0.01

Multicellular tumor spheroids (MCTS) generated from single cell suspension of continuous cell lines mimics in vivo tumor conditions [23]. The ability of a drug to impact tumor spheroid formation and spheroid invasion is an important indicator of drug sensitivity or resistance behavior of cancer cells found in solid tumors. Since aggressive local invasion is the hallmark of GBM, we examined the impact of antagonizing IL-8/CXCR1/2 with reparixin-l-lysine on tumor cell invasion. We observed a significant reduction in tumor spheroid growth and invasion of LN-18 cells within 24 h after treatment while the effect on U-87 MG cells was more gradual which became significant after 72 h (Fig. 6a and b).

## Discussion

Role of the IL-8 in tumor development and growth has been demonstrated in many cancers such as colon cancer, melanoma, prostate and ovarian cancer [24]. In the present investigation, we explored its role in tumor immunology, mitogenic action and invasion in GBM through in vitro study. The important findings of this study are that IL-8 promotes tumor growth by both autocrine and paracrine mechanism by binding to its receptors CXCR1 and CXCR2 as deduced from protein localization by immunohistochemistry. It may be weakly associated with microglial infiltration in GBM but has no role in recruitment or suppression of other immune cells, since no negative correlation with lymphocytic infiltration was seen. Presence of IL-8 and its receptors in GBM cells, both in tumor specimens and GBM cell lines indicates autocrine signalling which promotes GBM cell proliferation and invasion as indicated by cell viability and proliferation assay, clonogenic assays, and spheroid invasion assay. In addition to this IL-8 also promote neovascularization by both autocrine and paracrine mode in the form of vasculogenic mimicry by binding to its cognate receptor CXCR1. The expression of IL-8 and CXCR1 in GBM cells and expression of CXCR1 in tumor vasculature indicates a role in GBM angiogenesis.

Higher expression of IL-8 is associated with poor prognosis in cancers such as pancreatic cancer, breast cancer, ovarian cancer, lung cancer, colon, prostate, and bladder cancer [25]. In silico analysis of TCGA data for IL-8 expression and overall survival of GBM patients also displayed poor prognosis in patients with high IL-8 expression. The mechanism through which it accelerates GBM growth leading to poor prognosis could be its involvement in both angiogenesis and GBM cell proliferation and invasion. Previously, Zhang et al. had reported the role of IL-8 in GBM progression. In their study, they also observed high IL-8 expression in approximately 80% of GBM tissues. They further demonstrated that IL-8 in an autocrine manner promotes glioma progression by binding to its receptor CXCR1 [26]. Contrary to our results they did not observe any expression of CXCR2 in gliomas. Similarly, an autocrine signalling of IL-8 via CXCR1 has also been reported by Aceto et al. in HER2/HER3 positive breast cancer which led to higher invasive capacity of mammary cancer cells [27]. However, Yang et al. confirmed high expression of CXCR2 in higher grades of glioma tissues and reported positive correlation with the degree of malignancy and recurrence and also demonstrated that pharmacological blocking of CXCR2 significantly reduced the migration of glioma cells [28].

Counteracting neovascularization in tumors is widely considered an important strategy in limiting tumor growth [29]. Angiogenic factors that promote neovascularization and angiogenesis are the center of anti-angiogenic therapies. Although it is known that angiogenesis involves a complex interplay of various factors, VEGF has garnered maximum attention and has been used for therapeutic intervention. But the limited success in improving overall survival in GBM patients obliges to explore and target additional factors [30]. It has been shown that VEGF via autocrine signalling leads to the release of pro-inflammatory cytokines (CXCL1, IL-6, IL-8/CXCL8, and GRO-α/) via VEGFR2 in endothelial cells. Conversely, these cytokines induce expression of VEGF [31]. A recent report by Dwyer et al. provided evidence that glioblastoma secretome can induce angiogenic signals leading to disruption of VE-cadherin mediated cell-cell junctions and promoted endothelial permeability in brain microvessels [5]. The key component of this secretome was IL-8 which realized this effect via its receptor CXCR2. They also demonstrated activation of VEFGR2 by IL-8 even in absence of VEGF, thus displaying probable compensatory action of IL-8 in absence of VEGF which could be speculated as one of the reasons for patients receiving anti-VEGF therapy eventually becoming refractory to the treatment. In the present investigation, although we observed increased expression of IL-8 in GBM cells, however, localization of its receptor suggests a different interaction. Our observation was that CXCR1 was uniformly expressed in tumor-associated microvessels in GBM while CXCR2 expression was limited to tumor cells. Hence based on the localization pattern involvement of IL-8/CXCR1 in promoting angiogenesis is more likely. Further, we observed disruption of the tubular network in U-87MG cell line on neutralization with anti-CXCR1 antibody while no impact on the tubular network was seen on treatment with anti-CXCR2 which provides evidence in this regard. This study also provides an indication of IL-8/CXCR1 involvement in vasculogenic mimicry which is a non- classical mode of vascularization. Presence of CXCR1+/CD34- vessels in addition to CXCR1+/CD34+ vessels suggests that this axis may promote neovascularization by both classical and non-classical mechanisms, making it an attractive target for future anti-angiogenic therapies in GBM. To our knowledge, this is the first study showing the involvement of IL-8/CXCR1 axis in vascular mimicry in GBM.

Expression of IL-8 and its receptor CXCR2 in GBM cells made us speculate its probable mitogenic action. In vitro neutralization and pharmacological blocking of IL-8/CXCR1/2 led to significant reduction in GBM cell proliferation and viability. Further reduction in clonogenic survival provided evidence in support of mitogenic potential of IL-8. Mitogenic effect of IL-8 has been shown in other cancers as well, although detailed underlying mechanism driving cell proliferation has not yet been deciphered. Three primary downstream signalling cascades have been implicated which are activated on stimulation of CXCR1/2 by IL-8: phosphatidyl- inositol 3′ kinase/Akt (PI3K/Akt), phospholipase C/protein kinase C (PLC/PKC), and Ras/Raf/extracellular signal-regulated protein kinases 1 and 2 (Erk1/2). Other pathways which may also be involved are focal adhesion kinase, Rho, Rac, and Janus kinase/signal transducers and activators of transcription (JAK/STAT) [8].

3D tumor models such as tumor spheroids have rapidly gained importance due to their closer representation of in vivo tumor conditions compared to 2D cell culture models [32, 33]. Hence, we investigated the efficacy of the pharmacological antagonist of the CXCR1 and CXCR2 on 3D tumor spheroid growth and invasion. The treatment resulted in a significant impact on invasive capacity and overall size of these tumor spheroids which indicate important implications of targeting these axes for clinical purpose. Although many important cellular and microenvironment interactions are mimicked by the 3D tumor model, still, there are additional signalling parameters which are lacking in this system and may have a profound effect on the overall efficacy of this drug in vivo. Hence, we advocate further in vivo testing of this drug on orthotopic xenograft model which will strengthen its therapeutic candidature.

## Conclusion

**Fig. 7:**
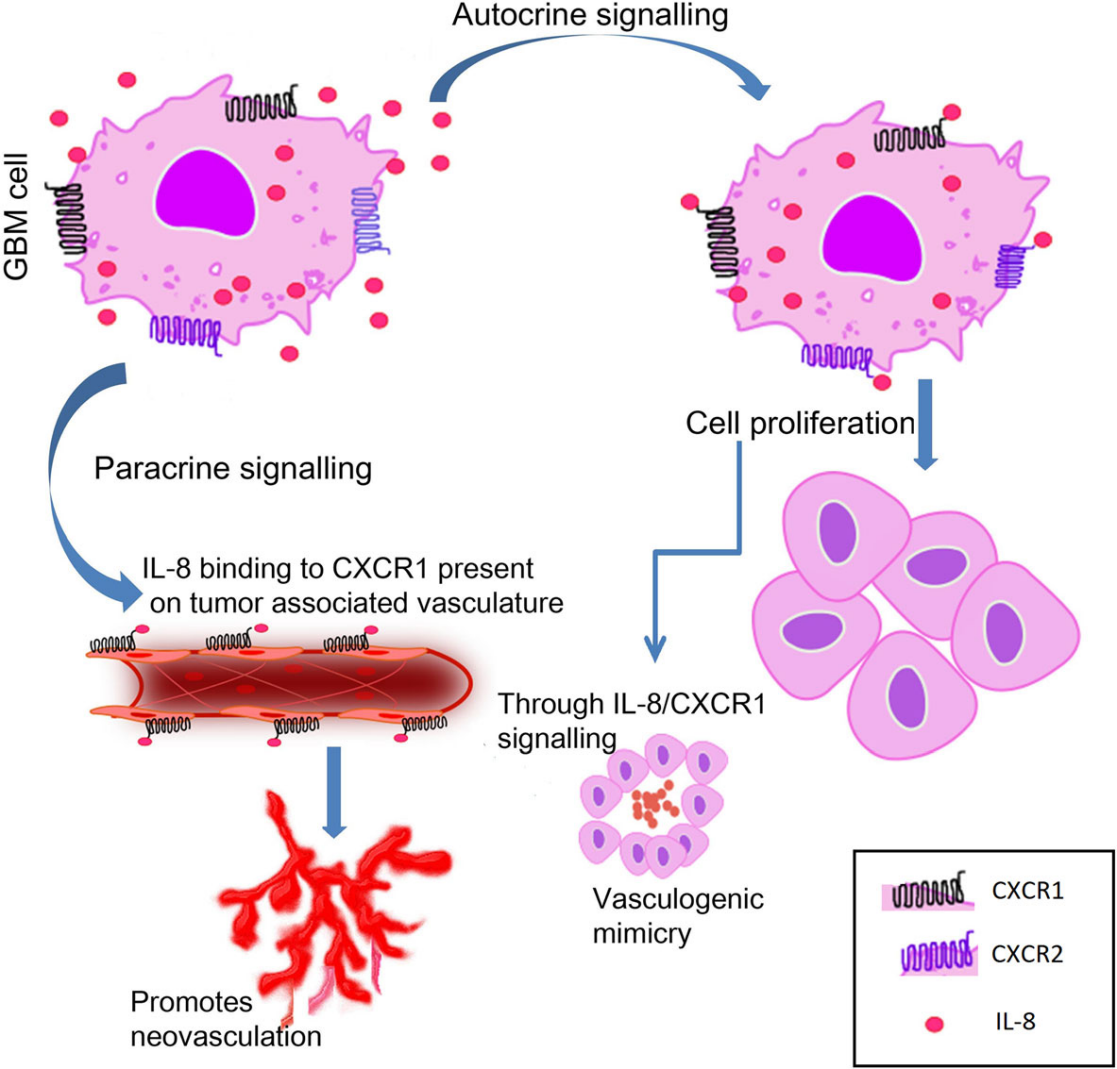
Hypothetical model depicting probable IL-8/CXCR1/2 signalling mechanism in GBM

IL-8/CXCR1/2 signalling is an important player in GBM biology which can influence GBM growth directly by promoting GBM cell proliferation and invasion and indirectly by promoting neovascularization by both classical and non-classical mechanism (Fig. 7). In vitro targeting of IL-8/CXCR1/2 axes provided notable evidence of their therapeutic potential which may be bolstered with further in vivo studies.

## Additional file

Additional file 1: **Table S1.** Gene wise primer sequence. (DOCX 15 kb)

## Abbreviations

GBM: Glioblastoma multiforme
IL-8: Interleukin-8
MTT: (3-(4, 5-Dimethylthiazol-2-yl)-2, 5-Diphenyltetrazolium Bromide)
VM: Vascular mimicry
