# Supplementary material for: IL-8/CXCR1/2 signalling promotes tumor cell proliferation, invasion and vascular mimicry in glioblastoma
Source: J Biomed Sci. 2018 Aug 8;25:62. doi: 10.1186/s12929-018-0464-y (PMC6081798; doi:10.1186/s12929-018-0464-y)
Supplement: Supplementary file 1 — Table S1. Gene wise primer sequence. (DOCX 15 kb) [file 12929_2018_464_MOESM1_ESM.docx]

**Table S1** Gene wise primer sequence:

| Gene Name | Primer sequence | Tm | Product length |
| --- | --- | --- | --- |
| CXCL8 | Forward, 5′-AGGGTTGCCAGATGCAATAC-3′;  Reverse, 5′-AAACCAAGGCACAGTGGAAC-3′ | 56°C | 420bp |
| CXCR1 | Forward, 5’-TCCTTTTCCGCCAGGCTTACCA-3’;  Reverse 5’- GGCACGATGAAGCCAAAGGTGT-3’ | 63°C | 127bp |
| CXCR2 | Forward, 5’- TCCGTCACTGATGTCTACCTGC-3’;  Reverse, 5’- TCCTTCAGGAGTGAGACCACCT-3’ | 62°C | 140bp |
| GAPDH | Forward, 5'-ACCACAGTCCATGCCATCAC-3  Reverse, 5'-TCCACCACCCTGTTGCTGT-3' | 56°C | 452bp |
